# The Entropy and Energy for Non-Mechanical Work at the Bose–Einstein Transition of a Harmonically Trapped Gas Using an Empirical Global-Variable Method

**DOI:** 10.3390/e26080658

**Published:** 2024-07-31

**Authors:** Marcos Miotti, Edmur Braga Martins, Michał Hemmerling, Vanderlei Salvador Bagnato

**Affiliations:** 1 São Carlos Institute of Physics, University of São Paulo, São Carlos 13566-590, Brazilvander@ifsc.usp.br (V.S.B.); 2 Biomedical Engineering Department, Texas A&M University, College Station, TX 77843, USA

**Keywords:** entropy, Bose–Einstein condensation, quantum gases, quantum thermal engines

## Abstract

Quantum thermal engines have received much attention in recent years due to their potential applications. For a candidate group, harmonically trapped gases under Bose–Einstein condensation (BEC), we see little investigation on the energy transference around that transition. Therefore, we present an empirical study with rubidium-87 gas samples in a magnetic harmonic trap. We developed an empirical equation of state model to fit to our experimental dataset, expressing the pressure parameter in terms of temperature, and six technical coefficients, functions of the volume parameter and the number of atoms. By using standard thermodynamic relations, we determine the system’s entropy, shown to be constant at the BEC transition, as expected. Being isentropic makes the BEC transition an energy source for non-mechanical work. Hence, we observed that the enthalpy at the BEC transition, at fixed values of the volume parameter, grows fairly linearly with the number of atoms. We fitted a linear function to that data, finding the specific enthalpy of the BEC transformation and the intrinsic enthalpic loss for BEC. We deem this study to be a step closer to practical quantum-based engines.

## 1. Introduction

With the discussions in mainstream media and the nearing deployment of operational quantum computers and other quantum-based technologies for actual applications, some branches of contemporary research on quantum thermodynamics have been devoted to achieving that goal, as classical thermodynamics was one of the major technoscientific causes for the First Industrial Revolution. In particular, the development of the so-called *quantum thermal engines* [1], which are machines running on thermal energy sources at a microscopic scale, has been a topic of interest in recent years. When it comes to thermal engines in classical thermodynamics, it is natural to think of devices using gases for combustion or work generation. For the latter, ultracold, quantum-degenerated gases seem promising candidates for building real quantum thermal engines. Indeed, Sur and Ghosh [2] have recently pointed out the advantages of using Fermi and Bose gases in quantum engines. Koch and co-workers [3] have actually implemented a thermal engine operating at a quantum phase transition crossover, showing that a phase transition in a bosonic–fermionic system is a viable energy source for work. In the particular case of bosonic systems, Eglinton and co-workers [4] have indicated a performance boost for a quantum engine running on a gas under Bose–Einstein condensation (BEC) under certain configurations. In the current year, Amette Estrada and co-workers [5] analyzed theoretically the role of interactions in the efficiency of thermal engines running on a harmonically confined gas under BEC, and Simmons and co-workers [6] have implemented a BEC-boosted, working fluid engine. However, after reviewing the recent literature, we believe that the field of weakly interacting gases under BEC in a harmonic potential still lacks an experimental investigation of the thermodynamic properties at the BEC transition, which we hope to address in this paper.

In the last year [7], we demonstrated theoretically and experimentally that the efficiency of Carnot cycles is always $1-T_{\mathrm{cold}}/T_{\mathrm{hot}}$ before, across and after BEC. For that end, we have used experimental data and the methodology first described in the corresponding author’s Master thesis [8], which relied on a formalism called the Global-Variable Method [9,10,11], an approach parallel to Local Density Approximation [12] in describing the thermodynamics of inhomogeneous quantum gases (remarkably, the harmonically trapped ones). Now, using the same data and approach, we present here a strictly experimental investigation on the BEC transition as an energy source for non-mechanical work, a topic that is seldom explored in the literature. Although more modern techniques are already available for producing gas samples under BEC at a constant density in box-like potentials [13], making thermodynamic analysis much easier, the simplicity and reliability of over forty years of expertise [14] on harmonically trapped ultracold gases and the existence of BEC laboratories with in-operation harmonic traps justify using the Global-Variable Method, as that mathematical formalism is already available.

The remainder of this paper is organized as follows: in Section 2, we review the Global-Variable Method, which was used for determining the thermodynamic quantities in our experiments; in Section 3, we describe briefly our apparatus for producing gases under BEC and the techniques used in our experiments. In Section 4, we present a full thermodynamic description of harmonically trapped gas samples across BEC, showing that the entropy is constant at the phase transition and investigating the energy available for non-mechanical work at it; in Section 5, we map out the steps for designing a quantum thermal engine running on a harmonically trapped gas based on Section 3 and Section 4, and in Section 6, we wrap up the results and their discussion in this paper.

## 2. Global-Variable Method

Differently from an ideal gas in a box-like potential, for which the confining volume (*V*) in space is well defined, an ideal gas in a harmonic potential $U_{h}\left(r\right)=\left(m/2\right)\left(\omega_{x}^{2}x^{2}+\omega_{y}^{2}y^{2}+\omega_{z}^{2}z^{2}\right)$ covers the whole space theoretically, which means that neither its volume nor its pressure (which forms a conjugate pair with volume) are well defined. However, it is still possible to find thermodynamic quantities for that kind of system, being one of few analytically solvable quantum many-body problems out there. By using the Bose–Einstein statistics in which the limit of the energy-level spacing is much lower than the thermal energy kT, with *T* being the temperature, the total number of atoms and the internal energy of an ideal gas in a harmonic potential are described as

(1) $$N\left(T,\bar{\omega }\right)={\left(\frac{kT}{\hbar \bar{\omega }}\right)}^{3}g_{3}\left(e^{\mu /(kT)}\right)$$

(2) $$\mathrm{and}\;\;E\left(T,\bar{\omega }\right)=3kT{\left(\frac{kT}{\hbar \bar{\omega }}\right)}^{3}g_{4}\left(e^{\mu /(kT)}\right)\;\;$$

respectively, in which $\bar{\omega }={\left(\omega_{x}\omega_{y}\omega_{z}\right)}^{1/3}$, μ is the chemical potential and $g_{\nu }\left(\xi \right)=\sum_{i=1}^{\infty }\xi^{i}/i^{\nu }$ is the polylogarithm (usually called “Bose function” among physicists). By making μ=0 in Equation (1), we find the *critical temperature* at which an ideal gas suffers BEC:

(3) $$T_{c}\left(N,\bar{\omega }\right)=\frac{\hbar \omega }{k}{\left(\frac{N}{g_{3}\left(1\right)}\right)}^{1/3}.$$

As *N* and *E* are both extensive quantities, this means that some quantity on the right-hand side of Equations (1) and (2) must also be extensive, and it is $\bar{\omega }$—which is logical, as the squared frequencies of a harmonic potential are directly proportional to how confining it is in each direction. On that account, let us define the quantity

(4) $$V\equiv 1/{\bar{\omega }}^{3},$$

and substitute it into Equation (3) to find

(5) $$T_{c}\left(N,V\right)=\frac{1}{{\left[g_{3}\left(1\right)\right]}^{1/3}}\frac{\hbar }{k}{\left(\frac{N}{V}\right)}^{1/3}.$$

As the critical temperature is a universal property, Equation (5) must hold in the thermodynamic limit (N→∞ and V→∞, thus n=N/V=constant), which is equivalent to weakening the harmonic potential into all directions, releasing atoms from their spatial confinement as their number grow. In that way, $\bar{\omega }\to 0$, which from Equation (4) makes V→∞ as N→∞; hence, N/V=constant in the thermodynamic limit, with Equation (5) holding true.

For its extensivity and analogy with volume, V defined in Equation (4) is called *the volume parameter*. Indeed, Romero and Bagnato [9,10,11] demonstrated together that V forms a conjugate pair with a quantity appropriately called *pressure parameter*, defined as

(6) $$P=\frac{2}{3V}\int_{V(U_{h})}n\left(r\right)U_{h}\left(r\right)\;d^{3}r,$$

in which *n* is the spatially distributed atomic density of the gas trapped by the harmonic potential $U_{h}$. That quantity is shown to be analogous to the hydrostatic pressure of a thermalized fluid at rest within a harmonic potential. Noteworthy, Equation (2) in terms of the global conjugate variables of work is written as

(7) E=3PV,

which is valid for both ideal and weakly interacting gases [15]. Hence, with the conjugate variables of mechanical work, it is possible to give a full thermodynamic description of a harmonically trapped gas. Our methods for implementing a harmonic trap for gas samples and measuring their density profiles is described in Section 3.

## 3. Thermodynamic Experiments

Our experimental setup for producing rubidium-87 (^87^Rb) gas samples under BEC is the well-known double magneto-optical trap (MOT) system, whose construction and operation are detailed in Chapter 4 of Ref. [8]. In summary, hot ^87^Rb gas is initially collected at the first MOT and transferred to the second MOT, in which the gas is cooled to millikelvins. The laser field is turned off once the second MOT is fully loaded, with atoms becoming confined in a purely magnetic trap, whose field is a “cigar-shaped” harmonic potential ($\omega_{x}\neq \omega_{y}=\omega_{z}$) with a non-zero minimum, ensuring the sample is in a single hyperfine state (always $|F=2,m_{F}=2\rangle$ in our case). From there, the sample is subjected to submicrokelvin cooling by radiofrequency evaporation, finally reaching the temperature–density conditions for BEC.

Once the gas sample is prepared in situ, at the bottom of the harmonic trap (whose frequencies are fixed, thus V from Equation (4) is constant) and at a steady, constant temperature state, its density is so high that it must be released from its confinement to freely expand adiabatically and isothermally before being imaged, a process known as the *time-of-flight technique* (TOF), which destroys the sample. The cross-section imaging of the gas sample allows us to determine its total number of atoms (*N*), in situ temperature (*T*) and expanded density profile $n_{\mathrm{TOF}}$. To recover the in situ density profile (*n*) from $n_{\mathrm{TOF}}$, we use the procedures of Castin–Dum regression [16] for the Bose-condensed fraction of atoms and You–Holland regression [17] for the non-Bose-condensed (thermal) fraction of atoms. With V and *n*, we determine the pressure parameter (P) using Equation (6). Therefore, we have the four required quantities (*N*, *T*, V and P) to describe the thermodynamics of the system, as we discuss in Section 4. We recapitulate the topic of designing thermodynamic experiments in Section 5.

## 4. Findings

**Figure 1:**
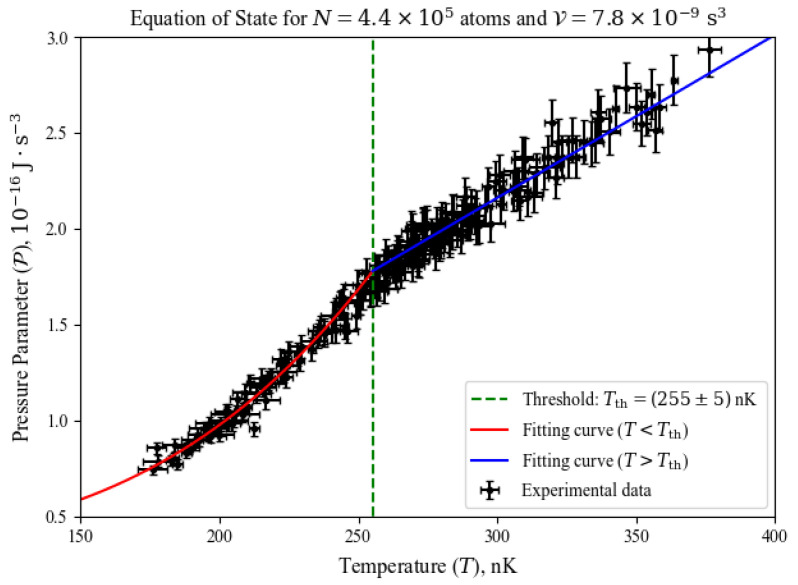
An example of the pressure parameter behavior as a function of temperature across the BEC transition for $V=7.8\times 10^{-9}\;s^{3}$ and $N=4.4\times 10^{5}$ atoms. The system smoothly transits from a linear behavior (the well-known Gay–Lussac’s law for classical gases) to a strongly nonlinear behavior (already indicated by Equation (2)) as temperature decreases. The blue, red and green lines represent the components of our empirical model for the equation of state, seen in Equation (8).

We have gathered a significantly large dataset of thermodynamic data, with twenty different values of *N* (ranging from $3.0\times 10^{5}$ to $5.5\times 10^{5}$ atoms), nineteen different values of V (ranging from $5.7\times 10^{-9}\;s^{3}$ to $9.7\times 10^{-9}\;s^{3}$), in the temperature range from $0.7T_{c}$ to $1.3T_{c}$. By fixing *N* and V, the usual behavior of P versus *T* is seen in Figure 1, which is the so-called the system’s *equation of state.*

From the overall behavior seen in Figure 1, we designed an empirical expression for the equation of state, as stated in Equation (8). For temperatures above the critical temperature (classical regime), any ideal gas is known to follow Gay–Lussac’s law, whose linear behavior is also seen in Figure 1. Therefore, we used a generalized linear function (with the linear coefficient $a_{4}$ and the slope $a_{3}$) to fit to the data in the classical regime. For temperatures below the critical temperature (quantum regime), a harmonically confined ideal gas has an internal energy described by Equation (2). From Equation (7), we generalized the power-of-4 function to include a linear coefficient ($a_{2}$), an additional exponent ($a_{1}$) to temperature, and a different factor ($a_{0}$) multiplying temperature, fitting it to our data in the quantum regime. As the measurements of the pressure parameter change smoothly between the quantum and classical regimes, the fitting parameter we call *threshold temperature* ($T_{\mathrm{th}}$) is defined at the point $P_{T<T_{c}}=P_{T>T_{c}}$. A brief description of those terms is listed below. For a deeper inspection of their physical meanings and values, refer to Chapter 6 in Ref. [8].

- $a_{0}$ shows very little dependency on *N* and V, becoming constant in the ideal case;
- $a_{1}$ is always negative, showing how the system deviates from the ideal $T^{4}$ behavior;
- $a_{2}$ is the zero-point pressure parameter of the system;
- $a_{3}$ is equivalent to the linear terms in Gay–Lussac’s law and the van der Waals equation;
- $a_{4}$ is related in meaning to the interaction term *a* in the van der Waals equation;
- $T_{\mathrm{th}}$ is at the intersecting point between the fitting functions; its value is not constrained by the actual value of the critical temperature of a system.

(8) $$P\left(T,V,N\right)=\left\{\begin{array}{cc} a_{0}\left(V,N\right)T^{4+a_{1}\left(V,N\right)}+a_{2}\left(V,N\right), & T<T_{\mathrm{th}}\left(V,N\right);\;T_{\mathrm{th}}≲T_{c}.\\ a_{3}\left(V,N\right)T+a_{4}\left(V,N\right), & T>T_{\mathrm{th}}\left(V,N\right). \end{array}\right.$$

From Equations (7) and (8), we have a complete mapping of the thermodynamics of a gas in a harmonic potential across the BEC transition. We demonstrate this by plotting P×V diagrams in Section 4.1 and T×S diagrams in Section 4.2. The latter will be particularly important in our analysis on the system’s enthalpy in Section 4.3.

### 4.1. P×V Diagrams and the BEC Transition

**Figure 2:**
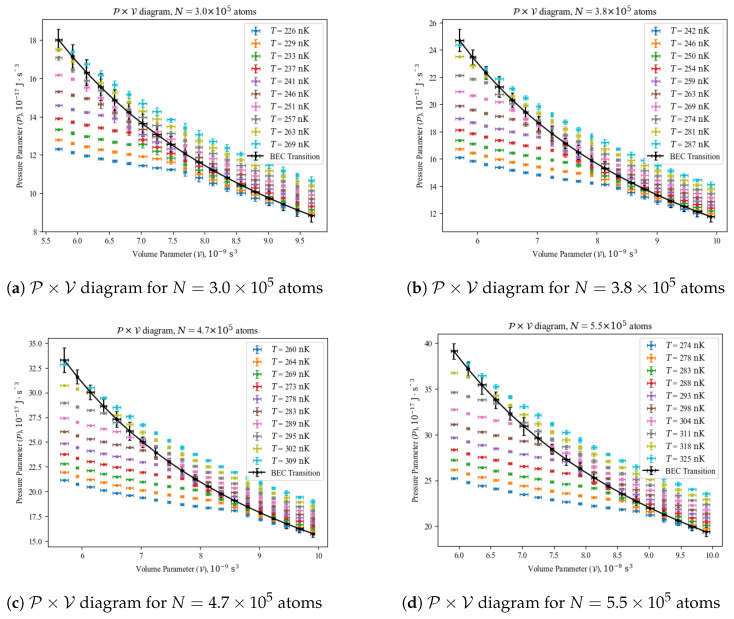
Selected pressure parameter (P) versus volume parameter (V) diagrams. Data points below black curve represent gas samples under BEC, whereas data points above black curve represent purely classical gas samples.

To illustrate the BEC transition in a clear manner, we plotted four P×V diagrams at a constant *N* in Figure 2. The black curve represents how the BEC transition behaves as V varies from one experiment set to another. Above the black curves, the gas samples are purely thermal or classical, well described by the Maxwellian distribution of energy states. Below the black curves, the gas samples have suffered BEC, with a fraction of the atoms populating the ground state of the harmonic trap, whereas the rest, called thermal atoms, are still described by the Maxwellian distribution.

As a reminder of Section 3, the measurements have been carried out by fixing the volume parameter of the harmonic trap (i.e., characterizing its frequencies) and collecting data for various temperatures and numbers of atoms. In our experimental setup, we did not have the flexibility of reliably changing the volume parameter of the harmonic trap without destroying the gas sample in that process. Although challenging, that procedure is indeed possible, as we will discuss later on in Section 5. The fact of the volume parameter being always constant in our measurements (i.e., no mechanical work is allowed in the system) influences our course of action in Section 4.2 and Section 4.3.

### 4.2. T×S Diagrams and the BEC Transition

**Figure 3:**
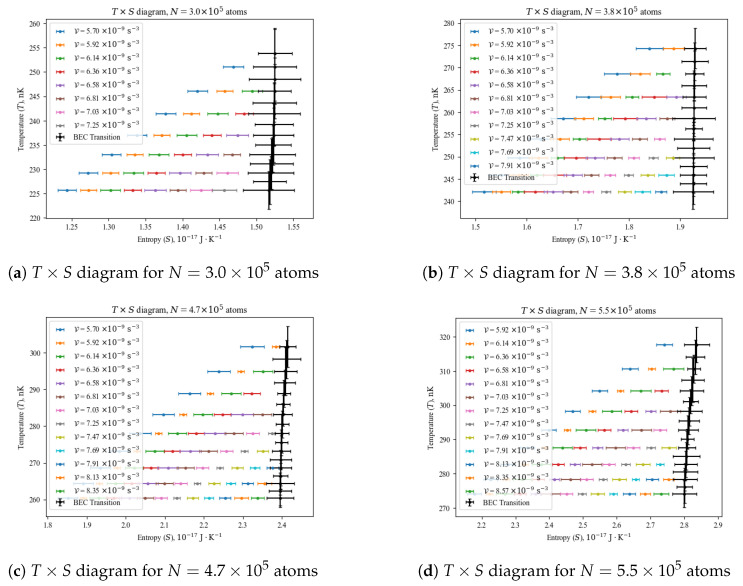
Selected temperature (*T*) versus entropy (*S*) diagrams. We show only the results for $T<T_{c}$, as the predictions with Equation (9) for $T>T_{c}$ fall within the uncertainty around the BEC transition. The horizontal spread ofthe data points is due to the varying values of V. Notice that the value of *S* at the BEC transition is constant for varying V, *T* and *N*, hence being an isentropic transformation.

By recalling Equation (7), we can use the relation E=TS−PV for fixed values of *N* to find the *actual* entropy of the system. From Equation (8), we obtain

(9) $$S\left(T,V,N\right)=\left\{\begin{array}{cc} 4V[a_{0}\left(V,N\right)T^{3+a_{1}\left(V,N\right)}+a_{2}\left(V,N\right)/T], & T<T_{\mathrm{th}}\left(V,N\right).\\ 4V[a_{3}\left(V,N\right)+a_{4}\left(V,N\right)/T], & T>T_{\mathrm{th}}\left(V,N\right). \end{array}\right.$$

whose plottings are shown in Figure 3 for four values of *N*. Notice that the points do not have error bars in the temperature axis, since *T* is an independent variable in both Equations (8) and (9), only used to generate the values and deviations for those equations of state. In the entropy axis, the magnitude of the error bars is significantly larger than that in Figure 2 due to error propagation in the operation S=4PV/T, especially at the BEC transition (black curve), as the critical temperature $T_{c}$ and the model’s threshold temperature $T_{\mathrm{th}}$ are both measured quantities, each having an associated error. For that reason, predictions with Equation (9) for $T>T_{c}$ are not shown in Figure 3, as they fall within the error bars at the transition.

The black curves indicating the BEC transition in Figure 3 are distinctive for having a constant entropy within the experimental error across the transition, in agreement with the fact that BEC is not associated with any latent heat [18]. Since each individual gas sample in our experiments has been probed at constant values of V, which prohibits mechanical work, we see that the BEC transition as a thermodynamic transformation could have been used as a source for non-mechanical work, as discussed in Section 4.3.

To support the results in Figure 3, let us recall that the BEC transition is an isentropic process (S=constant) for an ideal gas (which was our basis for designing the empirical equation of state in Equation (8) after all); from the internal energy in Equations (2) and (7), we find at the BEC transition (μ=0) that

(10) $$P=\frac{{\left(kT\right)}^{4}}{\hbar^{3}}g_{4}\left(1\right).$$

Now, the non-Bose-condensed (thermal) atomic fraction of an ideal, harmonically trapped gas is well known to be $N_{T}=N{\left(T/T_{c}\right)}^{3}$ for $T<T_{c}$, which may be written in the form $T=T_{c}{\left(N_{T}/N\right)}^{1/3}$. By raising that expression to the fourth power and substituting that and Equation (1) into Equation (10), we obtain

(11) $$P=\hbar \frac{g_{4}\left(1\right)}{{\left[g_{3}\left(1\right)\right]}^{4/3}}{\left(\frac{N_{T}}{V}\right)}^{4/3}\;\Rightarrow \;P{\left(\frac{V}{N_{T}}\right)}^{4/3}=\mathrm{constant}.$$

At the BEC transition ($T=T_{c}$), $N_{T}≅N$, which is constant; hence, Equation (11) becomes

(12) $$PV^{3/4}=\mathrm{constant}\;\mathrm{at}\;T=T_{c}\left(V\right).$$

Let us calculate the heat in a thermodynamic transformation from $\left(P_{1},V_{1}\right)$ to $\left(P_{2},V_{2}\right)$ over the level curve in Equation (12), which represents the BEC transition. From Equation (7) and $P=\mathrm{const}\cdot V^{-4/3}$, we have by the first law of thermodynamics that

(13) $$\begin{array}{cc} Q & =\Delta U+W=\Delta U+\int_{\left(P_{1},V_{1}\right)}^{\left(P_{2},V_{2}\right)}PdV\\ \; & =\left(3P_{2}V_{2}-3P_{1}V_{1}\right)+\int_{\left(P_{1},V_{1}\right)}^{\left(P_{2},V_{2}\right)}\left(\frac{\;\mathrm{const}\;}{V^{4/3}}\right)dV\\ \; & =3\cdot \;\mathrm{const}\;\left(\frac{V_{2}}{V_{2}^{4/3}}-\frac{V_{1}}{V_{1}^{4/3}}\right)-3\cdot \;\mathrm{const}\;{\left.\cdot \frac{1}{V^{1/3}}\right|}_{V=V_{1}}^{V_{2}}\\ Q & =0. \end{array}$$

In conclusion, the BEC transition as a thermodynamic process is adiabatic. When that process is performed in a reversible way, its entropy is also constant, which has already been shown by our experimental data in Figure 3.

### 4.3. $T_{c}S_{c}\times N$ Diagram: Energy for Non-Mechanical Work

**Figure 4:**
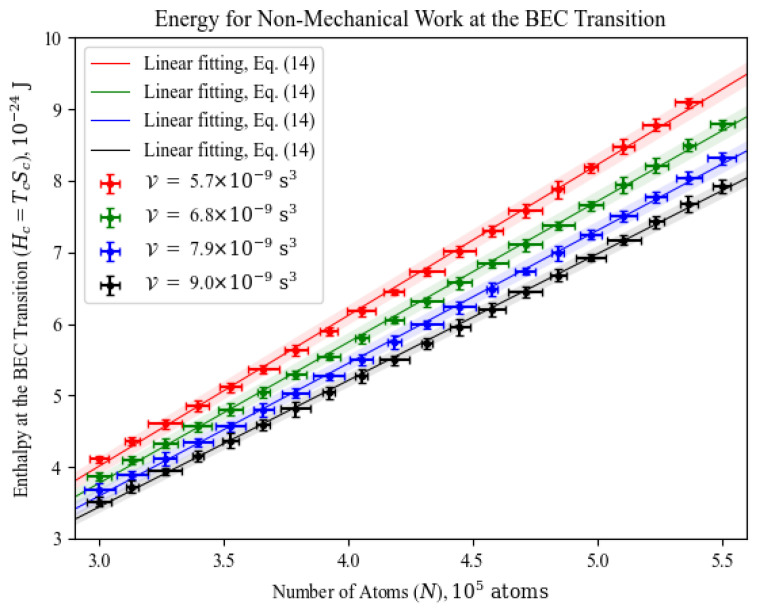
Constant volume parameter (V) curves of enthalpy at the BEC transition ($H_{c}=T_{c}S_{c}$) versus the number of atoms (*N*). Since the BEC transition is isentropic, the enthalpy there represents the useful energy that can be drawn from it for conducted non-mechanical work. To gain insight into the behavior of those curves, each group of constant V data points has been fitted by Equation (14).

By grouping our experimental data at their constant values of V, as they have been originally measured, we can determine the amount of energy available for non-mechanical work at the BEC transition as the system’s enthalpy, defined as H=E+PV, which from E=TS−PV yields $H_{c}=T_{c}S_{c}$ at the transition. The data points of $H_{c}$ versus *N* at selected values of V are seen in Figure 4. For the operation of a generalized thermal engine, the product $T_{c}S_{c}$ is more useful a quantity than the energy for mechanical work PV, which is “locked up” from use as the system’s volume parameter is held constant.

**Figure 5:**
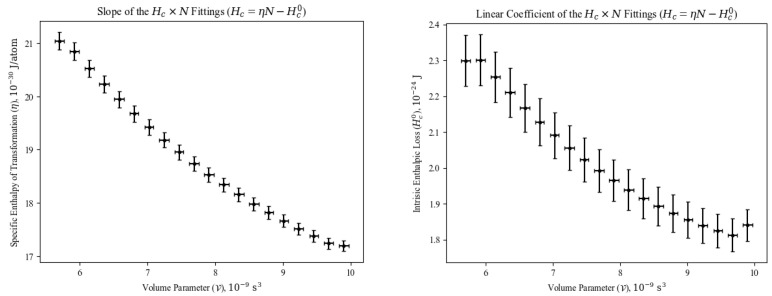
Results of fitting Equation (14) to data in Figure 4. Both terms are enthalpic costs required from large group of atoms to collectively undergo BEC, also giving us insight into typical energy values for non-mechanically moving atom from classical regime to quantum regime.

Although the curves in Figure 4 are not exactly straight lines, due to the nonlinear dependency of $T_{c}$ with V (already seen in Equations (3) and (5) for the ideal gas), it is visually clear that they can be approximated to a linear function, allowing us to gain insight into their behavior. Therefore, let us write a fitting line in the form

(14) $$H_{c}=\eta N-H_{c}^{0},$$

in which we call η the specific enthalpy of transformation for BEC, and $H_{c}^{0}$ the intrinsic enthalpic loss. The former represents the energy per atom for cooling or heating the gas across the BEC transition at constant V, i. e., without mechanical work. The latter is the amount of energy necessarily lost in adding zero to *N* atoms during the phase transition. We have fitted Equation (14) to all constant V curves (including those not shown in Figure 4), and the behavior of η and $H_{c}^{0}$ as functions of V is presented Figure 5.

There is a clear tendency in both the specific enthalpy of transformation (Figure 5a) and the intrinsic enthalpic loss under BEC (Figure 5b) to decrease as the system’s volume parameter increases, as the critical temperature decreases in that manner, as seen in Equations (3) and (5), all the while the entropy remains constant for all those values (see again Figure 3). By drawing an analogy with chemistry, Equation (14) can be seen as the variation in enthalpy when going from zero to *N* atoms, collectively reaching temperature–density conditions for BEC, whereas η is the enthalpic cost of moving one atom to that phase transition. At fixed values of V, the BEC transition is then a source of non-mechanical work, which could be used for moving atoms from high-energy states (classical, thermal gas) to the ground state (gas under BEC).

## 5. Performing Thermodynamic Cycles on Quantum Gases

As we discussed in Section 3 and Section 4, experiments in our setup have been conducted at constant values of V, given the limitation in safely transferring a sample from one value of V to another. However, as we have an empirical model in Equation (8) that allows us to give a full thermodynamic description of harmonically trapped, weakly interacting gases, we now desire to broaden this discussion by looking at varying V, a feasible feature in other experimental apparatuses around the world. In that scenario, it is possible to speculate about the potential implementation of thermodynamic transformations and cycles in gas samples under BEC, which consequently leads to ideas of quantum thermal engines. We list below the possible thermodynamic transformation under the Global-Variable Method for harmonically trapped gas samples. For this analysis, we consider that the number of atoms *N* is held constant in all of the listed processes.

- **Parametric–isochoric processes:** They follow the usual procedures mentioned in Section 3, with frequencies of the trapping potential measured and fixed. In those cases, all of the technical coefficients in Equation (8) are constant, and thus, P=P(T). By mapping the currents on the coils that create the magnetic fields of the harmonic potential, it is possible to vary the volume parameter between each experimental sequence to produce a harmonically trapped gas sample.
- **Isothermal processes:** The steady-state temperature of the gas sample in the harmonic trap is determined by its prior exposition to radiofrequency evaporation, which is a highly controllable and reproducible technique. Therefore, by mapping the exposure time and the strength of the radiofrequency signal, it is possible to obtain a gas sample at the same temperature with different volume parameters. In that case, Equation (8) becomes P=P(V). Varying V is viable by mapping the currents of the magnetic trap’s coils, as described in the previous item, or more easily by using optical dipole traps [19].
- **Parametric–isobaric processes:** From the equation of state in Equation (8), one can determine the temperature values necessary to obtain the same pressure parameter value at different volume parameters, with a combination of temperature and volume parameters from the two previously described processes. In those cases, the technical coefficients in Equation (8) are varying together with the temperature, but in such a way that P(T,V)=constant during the transformation.
- **Adiabatic processes:** By combining the first two processes described above, one can use Equation (9) to find a set of temperature and volume parameter (and consequently pressure parameter) values that yield S(T,V)=constant throughout the transformation. A natural choice in those cases is the BEC transition, which has been shown to be an isentropic process in Figure 3 whose critical temperature range can be estimated with the ideal gas $T_{c}$ in Equation (5), adding the correction terms of the Hartree–Fock approximation [20] for a more precise estimation.

To ensure that the transformations mentioned previously are indeed reversible, one could use a second gas sample of a different atomic species as a thermal reservoir. This allows one to willingly add or remove heat from the first gas sample while keeping its *N* constant. In that way, the thermal reservoir can be introduced or eliminated from the system without affecting the reversibility of any transformations on the working fluid.

We remark that any thermodynamic process or cycle can be theoretically designed by exploiting Equations (8) and (9), and experimentally implemented by following the procedures listed above. One can determine the work performed during a cycle or process by calculating the area (for a cycle) or the path integral (for a process) it describes in a P×V diagram, such as those seen in Figure 2. The heat input and output of a cycle are found by calculating the path integrals of each process forming that cycle in a T×S diagram, such as those in Figure 3; the sum of positive results yields the heat input, and the sum of negative results yields the heat output. With those procedures, one can determine the efficiency of any cycle by dividing its total work by its heat input. In this manner, we have a complete roadmap for implementing a thermal engine with a quantum gas.

## 6. Conclusions

We have presented here a full thermodynamic description of harmonically trapped gas samples with the Global-Variable Method in Section 4.1 and Section 4.2, which allowed us to find all thermodynamic potentials of such a system, in contrast with the standard Local Density Approximation method. By designing an empirical expression for the equation of state in Equation (8) and fitting it to a significantly large dataset of measurements, we have been able to obtain the system’s entropy directly and find that its value is constant at the BEC transition, confirming the known inexistence of latent heat for BEC.

Acknowledging that the volume parameter was always constant in our measurements and in some experiments with harmonic traps, thus prohibiting mechanical work, we determined the total energy available for non-mechanical work at the BEC transition by obtaining the system’s enthalpy as a function of the number of atoms for constant values of the volume parameter, as shown in Figure 4. Each constant-volume-parameter curve has been approximated by the linear function in Equation (14), allowing us the achieve the specific enthalpy of transformation across the BEC transition, and the inherent enthalpic cost that is required of the system when temperature–density conditions for BEC are matched. To our knowledge, this is first time such information is presented and discussed in the literature, showcasing the relevance of enthalpy for BEC.

We expanded our considerations by presenting in Section 5 how the already established techniques for producing and imaging ultracold gas samples in harmonic traps can be used to perform thermodynamic cycles in harmonic trap experiments across the globe. From our perspective, we are closer to systematically building thermal engines with quantum gases.

## Data Availability

Dataset available on request from the authors.

## References

[1] Mitchison M.T. (2019). Quantum thermal absorption machines: Refrigerators, engines and clocks. Contemp. Phys..

[2] Sur S., Ghosh A. (2023). Quantum Advantage of Thermal Machines with Bose and Fermi Gases. Entropy.

[3] Koch J., Menon K., Cuestas E., Barbosa S., Lutz E., Fogarty T., Busch T., Widera A. (2023). A quantum engine in the BEC–BCS crossover. Nature.

[4] Eglinton J., Pyhäranta T., Saito K., Brandner K. (2023). Thermodynamic geometry of ideal quantum gases: A general framework and a geometric picture of BEC-enhanced heat engines. New J. Phys..

[5] Amette Estrada J., Mayo F., Roncaglia A.J., Mininni P.D. (2024). Quantum engines with interacting Bose-Einstein condensates. Phys. Rev. A.

[6] Simmons E.Q., Sajjad R., Keithley K., Mas H., Tanlimco J.L., Nolasco-Martinez E., Bai Y., Fredrickson G.H., Weld D.M. (2023). Thermodynamic engine with a quantum degenerate working fluid. Phys. Rev. Res..

[7] Reyes-Ayala I., Miotti M., Hemmerling M., Dubessy R., Perrin H., Romero-Rochin V., Bagnato V.S. (2023). Carnot Cycles in a Harmonically Confined Ultracold Gas across Bose–Einstein Condensation. Entropy.

[8] Miotti M.P. (2021). Technical Thermodynamics of an Inhomogeneous Gas around the Bose-Einstein Transition Using the Global-Variable Method. Master’s Thesis.

[9] Romero-Rochín V. (2005). Equation of state of an interacting Bose gas confined by a harmonic trap: The role of the “harmonic” pressure. Phys. Rev. Lett..

[10] Romero-Rochín V., Bagnato V.S. (2005). Thermodynamics of an ideal gas of bosons harmonically trapped: Equation of state and susceptibilities. Braz. J. Phys..

[11] Romero-Rochín V. (2005). Thermodynamics and phase transitions in a fluid confined by a harmonic trap. J. Phys. Chem. B.

[12] Nascimbène S., Navon N., Jiang K., Chevy F., Salomon C. (2010). Exploring the Thermodynamics of a Universal Fermi Gas. Nature.

[13] Meyrath T., Schreck F., Hanssen J., Chuu C.S., Raizen M. (2005). Bose-Einstein Condensate in a Box. Phys. Rev. A.

[14] Ketterle W., Durfee D.S., Stamper-Kurn D. (1999). Making, probing and understanding Bose-Einstein condensates. arXiv.

[15] Shiozaki R.F., Telles G.D., Castilho P., Poveda-Cuevas F.J., Muniz S.R., Roati G., Romero-Rochin V., Bagnato V.S. (2014). Measuring the Heat Capacity in a Bose-Einstein Condensation Using Global Variables. Phys. Rev. A.

[16] Castin Y., Dum R. (1996). Bose-Einstein condensates in time dependent traps. Phys. Rev. Lett..

[17] You L., Holland M. (1996). Ballistic expansion of trapped thermal atoms. Phys. Rev. A.

[18] De Groot S., Hooyman G., Ten Seldam C. (1950). On the Bose-Einstein condensation. Proc. R. Soc. Lond. Ser. A Math. Phys. Sci..

[19] Kinoshita T., Wenger T., Weiss D.S. (2005). All-optical Bose-Einstein condensation using a compressible crossed dipole trap. Phys. Rev. A At. Mol. Opt. Phys..

[20] Pethick C.J., Smith H. (2008). Bose–Einstein Condensation in Dilute Gases.

